# The genomic landscape and clonal evolutionary trajectory of classical hairy cell leukemia

**DOI:** 10.1038/s41375-023-01823-1

**Published:** 2023-01-28

**Authors:** Luz Yurany Moreno Rueda, Dean Bryant, William J. Tapper, Nicola J. Weston-Bell, David C. Wedge, Naser Ansari-Pour, Surinder S. Sahota

**Affiliations:** 1 Tumour Immunogenetics Group, Cancer Sciences Unit, Faculty of Medicine, University of Southampton, Southampton, UK; 2 Genetic Epidemiology and Genomic Informatics Group, Human Genetics, Faculty of Medicine, University of Southampton, Southampton, UK; 3 Manchester Cancer Research Centre, University of Manchester, Manchester, UK; 4 MRC Molecular Haematology Unit, Weatherall Institute of Molecular Medicine, University of Oxford, Oxford, UK

**Keywords:** Cancer genomics, Oncogenes

In understanding clonal evolution in cancer, whole genome sequencing (WGS) provides unparalleled data that can be leveraged to derive tumor phylogenies. Specifically, estimates of the cancer cell fraction (CCF), that is the fraction of tumor cells bearing a set of somatic mutations, is highly informative by defining which mutations are clonal and are associated with the most recent common ancestor (MRCA) of all tumor cells, and which mutations occur subclonally as later events. By clustering all observed somatic mutations based on their CCF and subsequent phylogenetic tree inference, derived tumor phylogenies provide an even more detailed view of the tumor history than mere single point estimates of CCF of driver variants, as they determine the relative order of all events and infer evolutionary trajectories. These evolutionary trajectories inform which driver genes are early events in tumorigenesis, that dysregulate cellular molecular pathways critical to clonal proliferation.

HCL is a rare chronic B-cell lymphoproliferative disorder characterized by the presence of small tumor cells with hairy-like projections and membrane ruffles, which do not resemble any known peripheral normal B-cell subpopulation [1]. Typical classical HCL (HCLc) universally bears a coding *BRAF*^V600E^ mutation, making it a rare monogenic cancer. A large number of additional exonic mutations have been observed in HCLc, but none are pervasive across all tumors [2]. A conundrum, however, has remained that activating *BRAF* mutations also occur in benign tumors, including precursors to malignant carcinoma [3], to suggest that additional genetic drivers trigger malignant transformation. In HCLc, it is not yet known whether other driver mutations may arise in noncoding regions to cooperate with *BRAF*^V600E^ in driving tumorigenesis. To probe this, we carried out WGS in HCLc for the first time, and reconstructed subclonal genomic architecture and inferred evolutionary trajectories.

Our data provide, firstly, a comprehensive mapping of the whole genome in HCLc including noncoding regions to yield *BRAF*^V600E^ as the sole universal lesion in disease. Secondly, and importantly, clonal evolutionary analyses suggested that *BRAF*^V600E^ acts either alone or conjointly with 1 or 2 additional mutational drivers to initiate and promote malignant transformation in HCLc.

For the study, HCLc tumor and matched-germline T cells were highly purified (Supplementary Fig. 1) from spleen samples from 10 HCLc patients who underwent splenectomy and subjected to WGS. Full methods and computational analyses of WGS data, including somatic variant analysis, subclonal reconstruction and tumor phylogenies, are described in detail in the Supplementary Information. HCLc samples were kindly provided by Professor H. Kluin-Nelemans (University of Groningen, Netherlands) and used under local ethical approval. These samples are part of a historical sample bank. Splenectomy at the time was front line therapy. HCLc disease was diagnosed by clinical criteria, bone marrow and spleen histology, neoplastic cell morphology, cytochemical analysis and immunophenotype (CD11c/CD19/CD22/CD25) [4]. A summary of immunophenotype and mutation status of *IGHV* genes of HCLc cases are provided in Supplementary Table 1.

This report provides the first comprehensive description of the genome in HCLc *at disease presentation*.

The pattern of somatic coding mutations we identified was consistent with that previously reported from whole exome sequencing (Fig. 1) [2]. *BRAF*^V600E^ was the only mutation present in all cases as a clonal variant. Missense variants with potential deleterious effect were also identified in the transcription factor (TF) encoding *KLF2* in 2/10 tumors (p.S275N and p.S275I), previously reported in HCLc [5]. Two missense variants were further detected in *PRSS3* and *ANKRD30B* in 2/10 cases each (p.D95G and p.R94S - p.K388E and p.N392K respectively). *PRSS3* encodes an atypical isoform of trypsin that specifically activates the protease-activated receptor (PAR)-1 and triggers ERK phosphorylation increasing VEGF expression, promoting tumor progression [6]. The ubiquitin specific peptidase genes, *USP6* and *USP7*, and the dual specificity phosphatase genes, *DUSP2* and *DUSP27*, were also mutated in 1/10 case each (p.A64V and p.S133L - p.Q274X and p.S567N, respectively). Loss of function of *USP7* and *DUSP* genes may contribute with constitutive activation of MAPK signaling pathway, and may cooperate with *BRAF*^*V600E*^ in HCLc pathogenesis [7, 8]. Additional coding variants identified in individual HCLc cases are described in Supplementary Table 2.

**Fig. 1:**
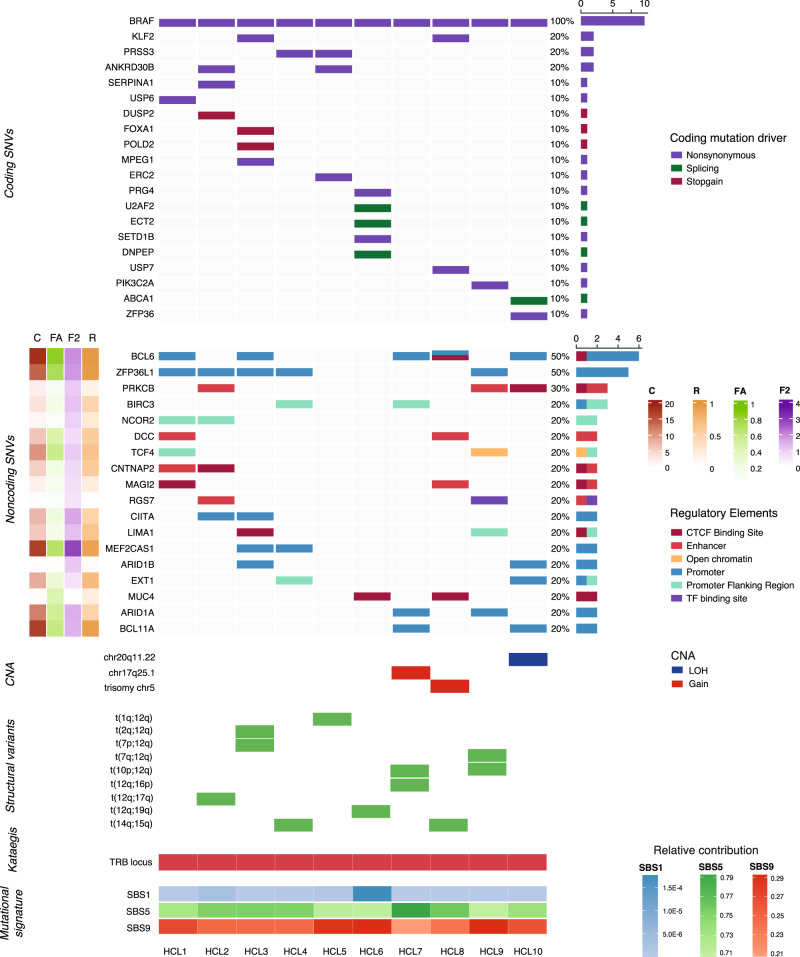
Mutational landscape of HCLc. *From top to bottom*: Driver coding somatic SNVs; noncoding somatic SNVs prioritized by the scoring algorithms CADD (C), REMM (R), FATHMM-MKL (FA) and  FunSeq2 (F2); CNA events and structural variants (translocations) with driver function; kataegis and mutational signatures identified in 10 HCLc tumors. In the first two panels, the scale bars indicate the total number of coding and noncoding SNVs identified in each gene in our cohort of HCLc samples.

The breadth of WGS data permitted identification of noncoding somatic variants in HCLc for the first time. Interestingly, the promoter regions of BCL6 transcription repressor (*BCL6*) and ZFP36 ring finger protein like 1 (*ZFP36L1*) were recurrently mutated in 50% of HCLc cases and prioritized by all noncoding scoring algorithms (Fig. 1 and Supplementary Fig. 2). These genes are known non-immunoglobulin targets for somatic hypermutation (SHM) in lymphomagenesis [9]. For instance, somatic variants in the 5’ regulatory region of *BCL6*, presumably produced by uncontrolled SHM, have been identified in different B-cell malignancies. These mutations disrupt the autoregulation circuit or the IRF4-mediated repression promoting its oncogenic functions [10], thus strongly suggesting its role in HCLc pathogenesis.

Chromosomal instability (CIN) is a hallmark of malignant transformation with copy number aberration (CNA) being ubiquitous across tumor types. Analysis of CNA showed a relatively stable genome in the HCLc cases (ploidy ≈2). We observed one large CNA (chr5 gain) and two focal CNAs (chr17q25.1 gain and chr20q11.22 LOH) (Fig. 1, Supplementary Fig. 3A). Although the role of these alterations in HCLc pathogenesis is not clear, this trisomy has been previously reported in 4/30 HCLc cases [11]. Interchromosomal translocations were the most frequent type of SV in this HCLc cohort (Supplementary Fig. 3B), with one translocation event at the histone methyltransferase *KMT2C* locus in HCL3. Importantly, recurrent inactivating coding variants in *KMT2C* have been previously identified in 15% (8/53) of a HCLc cohort, suggesting a potential function in the pathogenesis of this disease [12]. Recurrent translocations were also observed in the vicinity of the chr12q14.3 locus in 6 HCLc cases (Fig. 1). A common translocation, t(14q21.3;15q11.2), was also identified in 2 HCLc cases.

We searched all samples for kataegis, a phenomenon of localized hypermutation, and detected a novel striking signal in all samples within 7q34, spanning the T-cell receptor beta (*TRB*) locus (Fig. 1 and Supplementary Fig. 4). This is an interesting observation since such hypermutation in *TRB* appears novel among B-cell malignancies, and previously only reported in non-tumor settings [13].

Frequent mutations in *BCL6* prompted a wider examination of known AID off-target gene loci. Of 275 genes reported by Álvarez-Prado et al. [14], 51 gene loci were mutated in our HCLc cases, ranging from 4–13 genes per tumor. Eighteen genes were mutated ~2 kb downstream of the promoter, and 8 displayed mutations in AID motifs, including *B2M*, *CD83*, *GDI2*, *PIM1*, *RHOH* and *SWAP70*. Of the AID non-immunoglobulin target regions reported by Khodabakhshi et al. [9], 49 were identified as mutated in our HCLc cohort and 14 were AID motifs (Supplementary Fig. 5). Of these, somatic SNV detected in *KLF2* occurred in an AID motif suggesting an AID-induced mutation mechanism. AID targeted loci appear common in HCLc. Somatic mutational imprints in *BCL6* and *TRB* resemble SHM frequencies. They implicate GC origins of HCLc, and as CD27, a robust marker of GC memory, is absent in HCLc, it may suggest that transformation of HCLc occurs during early GC events, exiting prior to the acquisition of CD27. Ectopic SHM, however, cannot be excluded.

Mutational signature analysis provides insight into underlying mutagenic mechanisms. We identified single base substitution (SBS) COSMIC signatures SBS1, SBS5 and SBS9 in our HCLc cohort with similar relative contributions (Fig. 1) [15]. SBS1 and SBS5 are clock-like signatures observed across all tumor types and normal tissue samples, and SBS9 is associated with somatic hypermutation observed most prominently in lymphoproliferative disorders [15].

Central to the study, we further reconstructed the phylogenetic tree of each tumor based on the observed clonal cluster—representing the MRCA—and subclonal cluster(s) and assigned the driver variants to their respective clusters (Fig. 2). In all cases, *BRAF*^V600E^ occurred prior to subclonal diversification in the MRCA. In 6/10 cases, the MRCA also had one to two additional mutational drivers. Interestingly, all three CNA detected were secondary events and occurred subclonally (Fig. 2). All subclones had at least one driver mediating the post-MRCA clonal diversification. In two cases (HCL2 and HCL5), where multiple subclones were identified, linear evolution was inferred where the smaller subclone was nested within the larger subclone (Fig. 2). Interestingly, each subclone had its own unique driver, suggesting a multi-step process in HCLc tumor progression.

**Fig. 2:**
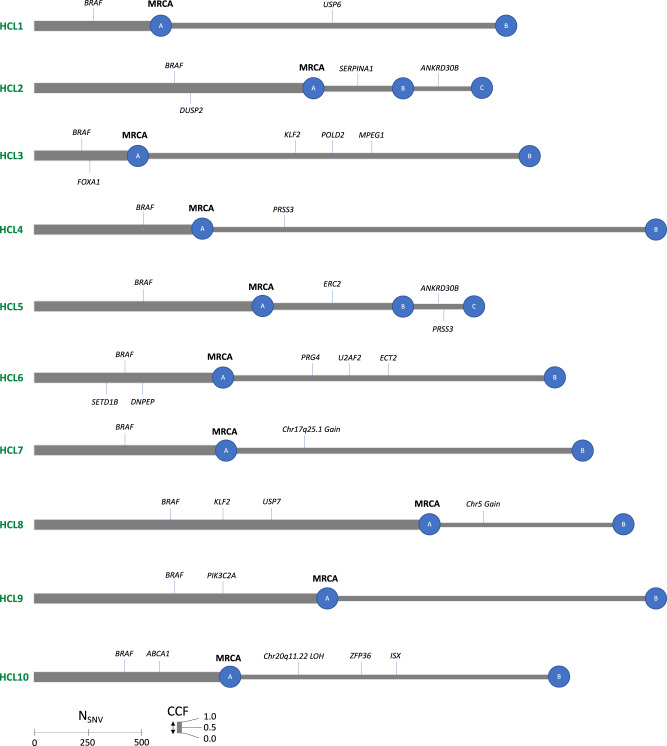
Tumor-level phylogenetic trees for 10 HCLc tumors. Branch lengths are proportional to the number of SNVs within the mutation cluster and branch thickness (height of gray bar) is proportional to the CCF of the cluster. Mutations that have occurred before the most recent common ancestor (MRCA) are carried by all tumor cells and define the clonal cluster of mutations. Each branch is annotated with known and candidate mutational and copy number drivers identified in each sample (the order of the drivers displayed on the branches is arbitrary). The mutation clusters are named alphabetically. The phylogeny in HCL2 and HCL5 can only be linear and not branching (subclonal clusters B and C with respect to the clonal cluster A) according to the pigeonhole principle.

Our findings conclude that *BRAF*^V600E^ alone may suffice to drive expansion of the ancestral clone (MRCA) to achieve malignant transformation, in at least a subset of HCLc. In separate pathways, *BRAF*^V600E^ may also require additional mutational drivers, that differ between tumors, for the MRCA to out-compete other cells and become clonal. These findings reveal heterogeneous genetic origins and subsequent subclonal diversification in HCLc disease.

## Supplementary information

Supplementary Data
