# Supplementary material for: The genomic landscape and clonal evolutionary trajectory of classical hairy cell leukemia
Source: Leukemia. 2023 Jan 28;37(4):929–33. doi: 10.1038/s41375-023-01823-1 (PMC10079533; doi:10.1038/s41375-023-01823-1)
Supplement: Supplementary file 1 — Supplementary Data [file 41375_2023_1823_MOESM1_ESM.docx]

**SUPPLEMENTAL DATA**

**Methods and Results**

**HCLc patient samples**

HCLc patient samples stored in liquid N_2_ were kindly provided by Professor H. Kluin-Nelemans (University of Groningen) and used under local ethical approval (NRES Committee South Central under REC M228/02/t).

Following splenectomy, splenic lymphocytes were disaggregated and stored in liquid N_2_ until use. Splenocytes from 10 HCLc patients were recovered and resuspended in complete RPMI medium (cRPMI) (RPMI 1640 (Gibco Invitrogen, Paisley, UK) containing 10% fetal calf serum (PAA Laboratories Ltd, UK), 1 mM sodium pyruvate MEM, 0.1 mM MEM non-essential amino acids, 2 mM L-glutamine (all Sigma-Aldrich, Poole, UK) and allowed to stabilize at 37 °C in 5% CO_2_ for 1 h prior to use. Viability was assessed by trypan blue exclusion. The expression of specific HCLc markers and the mutation status of *BRAF* and *IGHV* genes had previously been characterized in all samples [1].

**Purification of HCLc cells by fluorescence-activated cell sorting (FACS)**

Following stabilization at 37 °C for one hour, tumor cells and T cells from ten HCLc patient samples were purified using FACS. Splenocytes were washed with FACS buffer (1X PBS, 4 mM EDTA, 1% BSA, and 1 mM Azide) and pelleted for 5 min at 420 x g. Approximately 1x10^7^ cells were resuspended in 0.5 ml FACS buffer with 25 μl Human TruStain FcXTM (Biolegend, London, UK), and incubated for 10 min at room temperature. After blocking, splenocytes were stained with 40 μg/ml CD3-PE (clone: UCHT1, Biolegend, London, UK), 20 µg/ml CD11c-FITC (clone: Bu15, Biolegend, London, UK), 5 µg/ml CD103-PE/Cy7 (clone: Ber-ACT8, Biolegend, London, UK), and 2.5 µg/ml CD19-APC (clone: HIB19, Biolegend, London, UK) in 1ml FACS buffer. The mixture was incubated for 30 min at 4 °C. Following staining, cells were washed with 10 ml FACS buffer, centrifuged for 5 min at 420 x g, and resuspended in 1 ml FACS buffer for acquisition and sorting on the BD FACSAria. Data was analyzed using Flowjo (Treestar) software. Approximately, 6-12 million HCLc cells and 1-3 million T cells were sorted. The purity of sorted cells in each fraction was higher than 95% in all cases.

**DNA isolation and quantification**

Genomic DNA (gDNA) extraction from FACS-purified tumor and T cell populations was performed using QIAamp DNA mini kit (Qiagen, Manchester, UK), according to the manufacturer’s instructions. The DNA concentration was measured by using Qubit dsDNA BR assay kit and Qubit fluorometre, whilst 260/280 nm and 260/230 nm absorbance ratio were measured by NanoDrop ND1000 (Labtech, Uckfield, UK). gDNA concentrations ranged between 20 and 200 ng/μl and A260/A280 nm absorbance ratio > 1.80. gDNA samples were stored at -80 °C until use.

**Library preparation and WGS**

Library preparation and sequencing was performed by the Wellcome Trust Centre for Human Genetics in Oxford. gDNA from tumor and T cells were used for library preparation using NEBNext Ultra^TM^ DNA Library Prep kit (New England Biolabs, Hitchin, UK), according to manufacturer’s instructions. Sequencing was performed as 150 bp paired-end sequencing using Illumina HiSeq4000. The quality of the sequencing data was examined in the raw fastq files using FastQC. The reports showed high quality sequencing data with no sequences flagged as poor quality, absence of overrepresented sequences and adapter content, negligible level of N content and sequence duplication in all sample files. The sequencing was performed to a mean depth of 12×.

**Data analysis**

Reads from each library were mapped to the human reference genome GRCh37 using BWA-MEM (v0.7.12) producing SAM files [2]. Sorted BAM files for each sample were generated using SAMtools (v1.2.1) [3]. BAM files from the same sample obtained from different lanes were merged to generate one single BAM file per sample. Optical and PCR duplicates were flagged using Picard v1.97. The total number of reads in each BAM file was determined using SAMtools idxstat, with libraries containing between 2.23x10^8^ and 3.14x10^8^ reads, of which 99.8 to 99.9% mapped to GRCh37. From mapped reads, ~99% reads were pair aligned, 0.8% had the secondary alignment flags, and 0.1% were identified as singletons. From mapped paired reads, ~96% were found to be appropriately paired. About 4-5 million paired reads mapped to different chromosomes, but only ~2 million had a mapping quality score (MAPQ) >= 5. Singletons and improperly mapped paired reads were used for identification of possible somatic structural variation in tumor samples.

The analysis of the somatic variants in WGS data from matched tumor-normal HCLc samples was divided based on the nature of the variants, namely single-nucleotide variants (SNVs) and short insertions and deletions (indels), copy-number aberrations (CNAs) and structural variants (SVs).

The calling of somatic SNVs was conducted using CaVEMan (v1.10.4) [4]. These mutations were annotated using ANNOVAR [5]. The total number of somatically acquired single nucleotide variants (SNVs) called varied between 1884 - 3193. Maftools was used for the initial analysis and visualization of coding SNVs [6]. In addition to *BRAF*, genes with non-silent variants in at least two tumors or those with a non-silent variant observed in one tumor and also associated with tumorigenesis in the literature were considered as candidate driver genes. Potential driver genomic alterations located at cis-regulatory elements based on the Ensembl Regulatory Build database were identified using the Cancer Genome Interpreter (CGI) platform [7]. Prioritization of noncoding variants was performed by different scoring algorithms, including CADD[8], FATHMM-MKL[9], FunSeq2[10] and REMM[11], using the application SNPnexus[12]; and the recurrence across the HCLc cohort. The algorithm cgpPindel (v2.2.0) was used for the detection of small indels [13]. The regioneR v3.15 and karyoploteR v3.15 were used to identify and visualized kataegis events [14, 15]. Battenberg (v2.8), an allele-specific phase-informed subclonal copy number caller, was used for the detection of CNAs [16]. All CNA except those observed at the immunoglobulin loci (*IGK* at 2q, *IGH* at 14q and *IGL* at 22q) were considered as candidate copy number drivers. SVs, including large insertion and deletions, translocations, inversions and duplications, were identified using BRASS (v5.3.2) and annotated using GRASS [17]. Recurrence of translocation breakpoints across the cohort was used to identify hotspots of genomic rearrangements as candidate driver loci. The fitting of COSMIC mutational signatures identified in Alexandrov *et al* [18] was performed using *mmsig* (v0.0.0.9) [19] to minimize inter-bleeding of signatures in individual samples of the cohort.

To infer the subclonal architecture of each tumor, a Bayesian Dirichlet process algorithm was implemented (DPClust v2.2.2), to cluster somatic SNVs based on their cancer cell fraction[16, 20], which is the variant allele frequency (VAF) adjusted for CNA status of the locus and purity of the tumor sample. Variant clusters were identified as local peaks in the posterior variant density obtained from DPClust. In addition to the clonal cluster, the number of subclonal clusters and their respective mutation burden were also estimated. Tumor phylogenies were inferred using a previously described mathematical framework based on the pigeonhole principle [20]. WGS data have been deposited in the ArrayExpress database at EMBL-EBI ([www.ebi.ac.uk/arrayexpress](http://www.ebi.ac.uk/arrayexpress)) under accession number E-MTAB-12046.

**Code availability**

The code for somatic mutation identification can be at <https://github.com/cancerit/CaVEMan> (v1.10.4). The code for identification of small indels can be found at https://github.com/cancerit/cgpPindel (v2.2.0). The code for structural variants calling can be found at <https://github.com/cancerit/BRASS> (v5.3.2). The code for the mutational signature analysis can be found at <https://github.com/evenrus/mmsig> (v0.0.0.9). The code for the WGS subclonal copy number caller can be found at <https://github.com/Wedge-lab/battenberg> (v2.8). The code for the Dirichlet process-based methods for subclonal reconstruction of tumors can be found at <https://github.com/Wedge-lab/dpclust> (v2.2.2).

**Supplemental Figure 1**

**Supplemental Figure 1. Gating strategy used in cell sorting to purify HCLc tumor cells and germline T cells.** Splenocytes were stained with anti-CD19-APC, anti-CD3-PE, anti-CD11c-FITC and anti-CD103-PE/Cy7 prior to sorting on the FACSAria. In all cases, cells were initially gated on FSC-A vs FCS-H to remove clumping cells and then FSC-A vs SSC-A to exclude cell debris. Cells were selected followed by gating CD3^+^ CD19^-^ T cells and CD3^-^ CD19^+^ B cells. B cells were subsequently gated as CD11c^+^ and CD103^+^ to obtain high purity tumor cells. A) Pre-sort analysis of HCLc sample, which shows that 18.4% of cell population are T cells and 66.9% are tumor cells. B) Purified HCLc cells. C) Purified T cells.

**Supplemental Figure 2**

**Supplemental Figure 2. Location of noncoding somatic SNVs at the most recurrent mutated genomic regions in HCLc.** Lollipop plot of somatic SNVs identified at *BCL6* (A) and *ZFP36L1* (B) genes.

**Supplemental Figure 3**

**Supplemental Figure 3. The copy number aberration (CNA) and structural variation landscape in HCL.** A) Events in the positive direction are Gain events and in the negative direction are LOH events. The LOH events at 2q, 14q and 22q represents deletion event associated with *IGK*, *IGL* and *IGH* loci respectively. B) Circos plot of somatic structural variants.

**Supplemental Figure 4**

**Supplemental Figure 4.** **Regions of kataegis in HCLc.** A) Rainfall plot of somatic SNVs along the whole genome of a representative HCL case (HCL4). The distance between each mutation is represented in the *y* axis on a log base 10 scale. At the top, a density plot shows the number of somatic mutations in a window size of 10^5^. Each dot represents a variant and the color identifies the type of substitution: • C>A, • C>G, • C>T, • T>A, • T>C and • T>G. B) Lollipop plots of somatic SNVs identified at *TRB* loci in all HCLc cases.

**Supplemental Figure 5**

**Supplemental Figure 5. Mutated AID off-targets gene loci identified in HCLc cohort.** Somatic SNVs in AID motifs located at genomic regions previously identified as recurrent targets of SHM by Álvarez-Prado *et al* [21] and Khodabakhshi *et al* [22].

**Supplemental Table 1.**

**Table 1. Summary of immunophenotype and mutation status of IGHV genes of p.V600E BRAF-positive HCLc cases.** Adapted from Weston-Bell et al [1].

| **Case** | **HCL phenotype** | | | | **Surface Immunoglobulin (sIg) phenotype** | | | | | | ***IGHV* gene** | | |
| --- | --- | --- | --- | --- | --- | --- | --- | --- | --- | --- | --- | --- | --- |
|  | **CD19** | **CD11c** | **CD27** | **CD103** | **A** | **D** | **G** | **M** | **K** | **L** | ***IGHV*** | **% Homo** | **ICH** |
| HCL1 | + | + | ND | + | - | - | +++ | ++ | +++ | - | 3-30 | 96.7 | - |
| HCL2 | + | + | - | + | - | - | +++ | ++ | +++ | - | 3-30 | 96.1 | - |
| HCL3 | + | + | - | + | +++ | - | +++ | + | ++ | +++ | 3-15 | 92.2 | + |
| HCL4 | + | + | - | + | + | +++ | ++ | + | ++ | +++ | 5-51 | 98.6 | + |
| HCL5 | + | + | - | + | - | +++ | +++ | +++ | +++ | - | 3-23 | 96.5 | + |
| HCL6 | + | + | - | + | - | +++ | +++ | +++ | +++ | - | 3-30 | 92.7 | - |
| HCL7 | + | + | - | + | ++ | - | +++ | + | - | +++ | 1-46 | 95.1 | + |
| HCL8 | + | + | - | + | + | - | +++ | + | +++ | - | 3-20 | 94.8 | - |
| HCL9 | + | + | - | + | + | +++ | +++ | ++ | ++ | +++ | 3-33 | + |  |
| HCL10 | + | + | - | + | - | +++ | - | - | - | ++ | 3-30 | 94.1 | ND |

The percentage of hairy cells expressing each sIg isotype is represented as: ‘-‘ <10% cells, ‘+’ 10 – 50% cells, ‘++’ 50 – 90% cells, ‘+++’ >90% cells. ICH denotes ‘Intraclonal Heterogeneity*’* in *IGHV* gene sequences. ND denotes ‘Not Determined*’*.

**Supplemental Table 2**

**Table 2. Coding somatic mutations in 10 HCLc cases.**

| **Sample** | **Genetic coodinates hg19** | **Gene** | **Subclonal fraction** | **Location** | **Mutation type** | **Ref** | **Alt** | **AA change** |
| --- | --- | --- | --- | --- | --- | --- | --- | --- |
| HCL2 | 11:102991531 | DYNC2H1 | 1.382 | 1.067 | nonsynonymous SNV | G | C | Q416H |
|  | 3:63965726 | ATXN7 | 1.166 | 1.067 | nonsynonymous SNV | T | A | L67H |
|  | 7:140453136 | BRAF | 0.588 | 0.545 | nonsynonymous SNV | A | T | V600E |
|  | 1:2341870 | PEX10 | 0.691 | 0.612 | nonsynonymous SNV | A | T | W45R |
|  | 17:5035726 | USP6 | 0.173 | 0.612 | nonsynonymous SNV | C | T | A64V |
| HCL3 | 1:82456341 | ADGRL2 | 1.000 | 0.986 | nonsynonymous SNV | G | A | D1242N |
|  | 11:76916586 | MYO7A | 1.143 | 0.986 | nonsynonymous SNV | G | A | V1854M |
|  | 16:76513435 | CNTNAP4 | 0.769 | 0.986 | nonsynonymous SNV | G | A | E499K |
|  | 2:187514597 | ITGAV | 0.750 | 0.986 | nonsynonymous SNV | G | T | R425L |
|  | 2:96809687 | DUSP2 | 0.727 | 0.986 | stopgain | G | A | Q274X |
|  | 3:190576742 | GMNC | 0.667 | 0.986 | nonsynonymous SNV | G | A | S81F |
|  | 3:32776449 | CNOT10 | 1.500 | 0.986 | nonsynonymous SNV | G | A | E499K |
|  | 5:131280848 | MEIKIN | 0.750 | 0.986 | splicing | C | T | . |
|  | 6:80196785 | LCA5 | 1.067 | 0.986 | nonsynonymous SNV | T | A | D677V |
|  | 7:140453136 | BRAF | 0.588 | 0.545 | nonsynonymous SNV | A | T | V600E |
|  | 8:144810688 | FAM83H | 0.889 | 0.986 | nonsynonymous SNV | C | T | G315R |
|  | 14:94845827 | SERPINA1 | 0.615 | 0.587 | nonsynonymous SNV | C | G | E347Q |
|  | 14:94845829 | SERPINA1 | 0.615 | 0.587 | nonsynonymous SNV | G | C | T346R |
|  | 14:64066303 | WDR89 | 0.364 | 0.493 | nonsynonymous SNV | T | G | S120R |
|  | 18:14764026 | ANKRD30B | 0.300 | 0.493 | nonsynonymous SNV | A | G | K388E |
| HCL5 | 12:121176943 | ACADS | 1.250 | 1.024 | nonsynonymous SNV | G | A | E344K |
|  | 12:57587358 | LRP1 | 1.250 | 1.024 | nonsynonymous SNV | G | A | R2565H |
|  | 13:41767996 | KBTBD7 | 1.692 | 1.024 | nonsynonymous SNV | C | T | R133H |
|  | 14:38061368 | FOXA1 | 1.091 | 1.024 | stopgain | G | T | Y207X |
|  | 22:40082106 | CACNA1I | 1.200 | 1.024 | nonsynonymous SNV | A | G | H2088R |
|  | 10:12111177 | DHTKD1 | 0.476 | 0.629 | nonsynonymous SNV | C | T | R49C |
|  | 10:23728495 | OTUD1 | 0.750 | 0.629 | stopgain | C | T | Q37X |
|  | 10:8006872 | TAF3 | 0.533 | 0.629 | nonsynonymous SNV | G | C | D467H |
|  | 11:58978866 | MPEG1 | 0.556 | 0.629 | nonsynonymous SNV | C | T | M491I |
|  | 11:58978868 | MPEG1 | 0.667 | 0.629 | nonsynonymous SNV | T | A | M491L |
|  | 11:58978873 | MPEG1 | 0.750 | 0.629 | nonsynonymous SNV | T | G | N489T |
|  | 12:76424981 | PHLDA1 | 0.727 | 0.629 | nonsynonymous SNV | G | A | L181F |
|  | 15:45694643 | SPATA5L1 | 0.625 | 0.629 | nonsynonymous SNV | G | A | D6N |
|  | 15:83335562 | AP3B2 | 0.923 | 0.629 | nonsynonymous SNV | G | A | R565C |
|  | 16:5040888 | SEC14L5 | 0.706 | 0.629 | nonsynonymous SNV | G | A | V156I |
|  | 19:16436775 | KLF2 | 0.750 | 1.008 | nonsynonymous SNV | G | A | S275N |
|  | 19:23927204 | ZNF681 | 0.400 | 0.629 | nonsynonymous SNV | G | C | T383R |
|  | 20:36869214 | KIAA1755 | 0.889 | 0.629 | nonsynonymous SNV | T | C | E440G |
|  | 22:46777752 | CELSR1 | 0.875 | 0.629 | nonsynonymous SNV | C | T | R2360H |
|  | 5:110843098 | STARD4 | 0.769 | 0.629 | nonsynonymous SNV | T | C | T12A |
|  | 6:30653382 | PPP1R18 | 0.462 | 0.629 | nonsynonymous SNV | C | G | E138D |
|  | 7:140453136 | BRAF | 0.588 | 0.545 | nonsynonymous SNV | A | T | V600E |
|  | 7:44161640 | POLD2 | 0.824 | 0.629 | stopgain | G | A | Q5X |
|  | 7:5568938 | ACTB | 0.333 | 0.629 | nonsynonymous SNV | G | A | H73Y |
|  | 8:125740187 | MTSS1 | 0.571 | 0.629 | nonsynonymous SNV | C | T | V4M |
| HCL6 | 13:101735527 | NALCN | 1.125 | 0.855 | nonsynonymous SNV | T | A | K1173N |
|  | 19:44891716 | ZNF285 | 1.000 | 0.855 | nonsynonymous SNV | G | C | P76A |
|  | 4:96166153 | UNC5C | 1.111 | 0.855 | nonsynonymous SNV | T | G | K306N |
|  | 6:54806759 | FAM83B | 1.143 | 0.855 | nonsynonymous SNV | G | A | R997Q |
|  | 7:140453136 | BRAF | 0.588 | 0.545 | nonsynonymous SNV | A | T | V600E |
|  | 7:99956594 | PILRB | 1.143 | 0.855 | nonsynonymous SNV | C | T | R116W |
|  | 1:38411503 | INPP5B | 0.545 | 0.611 | nonsynonymous SNV | C | T | C26Y |
|  | 13:114058326 | LOC101928841 | 0.250 | 0.611 | nonsynonymous SNV | G | C | H1393Q |
|  | 17:61776028 | LIMD2 | 0.615 | 0.611 | nonsynonymous SNV | G | A | P90S |
|  | 19:50412035 | NUP62 | 0.800 | 0.611 | nonsynonymous SNV | G | C | L344V |
|  | 9:33797929 | PRSS3 | 0.235 | 0.611 | nonsynonymous SNV | G | C | R94S |
|  | 9:33797931 | PRSS3 | 0.364 | 0.611 | nonsynonymous SNV | A | G | D95G |
| HCL7 | 17:76510935 | DNAH17 | 0.884 | 1.096 | nonsynonymous SNV | G | A | T1345M |
|  | 18:68309789 | GTSCR1 | 1.237 | 1.096 | stopgain | G | T | C91X |
|  | 9:136521705 | DBH | 1.100 | 1.096 | stopgain | C | T | Q499X |
|  | 3:56330188 | ERC2 | 0.562 | 0.685 | nonsynonymous SNV | C | A | M311I |
|  | 18:14764040 | ANKRD30B | 0.206 | 0.582 | nonsynonymous SNV | T | G | N392K |
|  | 9:33797929 | PRSS3 | 0.235 | 0.611 | nonsynonymous SNV | G | C | R94S |
|  | 9:33797931 | PRSS3 | 0.364 | 0.611 | nonsynonymous SNV | A | G | D95G |
| HCL9 | 1:160136802 | ATP1A4 | 1.273 | 1.014 | nonsynonymous SNV | C | G | R431G |
|  | 1:248309345 | OR2M5 | 1.000 | 1.014 | nonsynonymous SNV | G | A | R299K |
|  | 11:62761095 | SLC22A8 | 1.200 | 1.014 | nonsynonymous SNV | T | G | T321P |
|  | 12:122260906 | SETD1B | 1.143 | 1.014 | nonsynonymous SNV | T | C | L1431P |
|  | 17:71232390 | C17orf80 | 1.143 | 1.014 | nonsynonymous SNV | C | T | H257Y |
|  | 2:10101179 | GRHL1 | 1.000 | 1.014 | nonsynonymous SNV | A | G | S95G |
|  | 2:210837971 | UNC80 | 1.000 | 1.014 | nonsynonymous SNV | C | A | S2789Y |
|  | 2:220247851 | DNPEP | 1.143 | 1.014 | splicing | A | G | . |
|  | 7:140453136 | BRAF | 0.588 | 0.545 | nonsynonymous SNV | A | T | V600E |
|  | 1:186277208 | PRG4 | 0.727 | 0.592 | nonsynonymous SNV | T | C | L652P |
|  | 16:56509971 | OGFOD1 | 0.800 | 0.592 | nonsynonymous SNV | C | A | P457T |
|  | 19:56171541 | U2AF2 | 0.800 | 0.592 | splicing | A | C | . |
|  | 3:172469939 | ECT2 | 0.667 | 0.592 | splicing | G | T | . |
| HCL10 | 1:34003079 | CSMD2 | 0.909 | 1.038 | nonsynonymous SNV | G | C | C3110W |
|  | 16:81078523 | ATMIN | 1.091 | 1.038 | nonsynonymous SNV | G | C | S651T |
|  | 17:4784456 | MINK1 | 1.429 | 1.038 | nonsynonymous SNV | C | G | I70M |
|  | 5:41160378 | C6 | 1.059 | 1.038 | nonsynonymous SNV | T | G | K517T |
|  | 7:140453136 | BRAF | 0.588 | 0.545 | nonsynonymous SNV | A | T | V600E |
| HCL11 | 11:55798847 | OR5AS1 | 1.092 | 1.008 | nonsynonymous SNV | G | A | R318H |
|  | 16:9015090 | USP7 | 1.031 | 1.008 | nonsynonymous SNV | G | A | S133L |
|  | 19:16436775 | KLF2 | 0.750 | 1.008 | nonsynonymous SNV | G | T | S275I |
|  | 22:38494114 | BAIAP2L2 | 0.687 | 1.008 | nonsynonymous SNV | A | C | W141G |
|  | 7:140453136 | BRAF | 0.588 | 0.545 | nonsynonymous SNV | A | T | V600E |
|  | 8:145677891 | CYHR1 | 0.916 | 1.008 | nonsynonymous SNV | G | A | T225M |
| HCL13 | 11:17140796 | PIK3C2A | 1.250 | 1.005 | nonsynonymous SNV | G | C | L591V |
|  | 13:25744097 | AMER2 | 0.909 | 1.005 | nonsynonymous SNV | G | A | A554V |
|  | 15:38228595 | TMCO5A | 0.875 | 1.005 | nonsynonymous SNV | C | T | T24M |
|  | 2:21361740 | TDRD15 | 1.286 | 1.005 | nonsynonymous SNV | A | T | L467F |
|  | 2:31215776 | GALNT14 | 0.947 | 1.005 | nonsynonymous SNV | A | C | L76R |
|  | 22:41257740 | DNAJB7 | 0.889 | 1.005 | nonsynonymous SNV | C | T | G87S |
|  | 5:140589119 | PCDHB12 | 1.200 | 1.005 | nonsynonymous SNV | G | A | A214T |
|  | 6:71266477 | FAM135A | 1.097 | 1.005 | nonsynonymous SNV | T | G | V1189G |
|  | 6:84836233 | CEP162 | 1.000 | 1.005 | splicing | T | C | . |
|  | 9:131588341 | SPOUT1 | 1.400 | 1.005 | nonsynonymous SNV | C | T | R200Q |
|  | 2:179483079 | TTN | 0.462 | 0.488 | nonsynonymous SNV | C | A | K6637N |
|  | 7:140453136 | BRAF | 0.588 | 0.545 | nonsynonymous SNV | A | T | V600E |
| HCL14 | 1:167096068 | DUSP27 | 0.889 | 1.005 | nonsynonymous SNV | G | A | S567N |
|  | 12:8629924 | CLEC6A | 1.273 | 1.005 | nonsynonymous SNV | A | G | H135R |
|  | 7:140453136 | BRAF | 0.588 | 0.545 | nonsynonymous SNV | A | T | V600E |
|  | 7:45123786 | NACAD | 2.000 | 1.005 | nonsynonymous SNV | G | T | L665I |
|  | 9:107556793 | ABCA1 | 1.200 | 1.005 | splicing | T | A | . |
|  | 9:39085821 | CNTNAP3 | 1.000 | 1.005 | splicing | C | A | . |
|  | 11:48387279 | OR4C5 | 0.571 | 0.629 | nonsynonymous SNV | G | A | R247W |
|  | 13:111109534 | COL4A2-AS2 | 0.857 | 0.629 | nonsynonymous SNV | G | C | Q180E |
|  | 14:21498855 | TPPP2 | 0.600 | 0.629 | nonsynonymous SNV | A | T | I39F |
|  | 19:39898441 | ZFP36 | 0.667 | 0.629 | nonsynonymous SNV | C | T | S34F |
|  | 22:35463115 | ISX | 0.800 | 0.629 | nonsynonymous SNV | G | A | G12D |

**Supplemental References**

1. Weston-Bell, N.J., F. Forconi, H.C. Kluin-Nelemans and S.S. Sahota. Variant B cell receptor isotype functions differ in hairy cell leukemia with mutated BRAF and IGHV genes*.* *PLoS One*, 2014. **9**(1): p. e86556.

2. Li, H. and R. Durbin. Fast and accurate short read alignment with Burrows-Wheeler transform*.* *Bioinformatics*, 2009. **25**(14): p. 1754-60.

3. Li, H., B. Handsaker, A. Wysoker, T. Fennell, J. Ruan, N. Homer*, et al.* The Sequence Alignment/Map format and SAMtools*.* *Bioinformatics*, 2009. **25**(16): p. 2078-9.

4. Jones, D., K.M. Raine, H. Davies, P.S. Tarpey, A.P. Butler, J.W. Teague*, et al.* cgpCaVEManWrapper: Simple Execution of CaVEMan in Order to Detect Somatic Single Nucleotide Variants in NGS Data*.* *Curr Protoc Bioinformatics*, 2016. **56**: p. 15 10 1-15 10 18.

5. Wang, K., M. Li and H. Hakonarson. ANNOVAR: functional annotation of genetic variants from high-throughput sequencing data*.* *Nucleic Acids Res*, 2010. **38**(16): p. e164.

6. Mayakonda, A., D.C. Lin, Y. Assenov, C. Plass and H.P. Koeffler. Maftools: efficient and comprehensive analysis of somatic variants in cancer*.* *Genome Res*, 2018. **28**(11): p. 1747-1756.

7. Tamborero, D., C. Rubio-Perez, J. Deu-Pons, M.P. Schroeder, A. Vivancos, A. Rovira*, et al.* Cancer Genome Interpreter annotates the biological and clinical relevance of tumor alterations*.* *Genome Med*, 2018. **10**(1): p. 25.

8. Kircher, M., D.M. Witten, P. Jain, B.J. O'Roak, G.M. Cooper and J. Shendure. A general framework for estimating the relative pathogenicity of human genetic variants*.* *Nat Genet*, 2014. **46**(3): p. 310-5.

9. Rogers, M.F., H.A. Shihab, M. Mort, D.N. Cooper, T.R. Gaunt and C. Campbell. FATHMM-XF: accurate prediction of pathogenic point mutations via extended features*.* *Bioinformatics*, 2018. **34**(3): p. 511-513.

10. Fu, Y., Z. Liu, S. Lou, J. Bedford, X.J. Mu, K.Y. Yip*, et al.* FunSeq2: a framework for prioritizing noncoding regulatory variants in cancer*.* *Genome Biol*, 2014. **15**(10): p. 480.

11. Smedley, D., M. Schubach, J.O.B. Jacobsen, S. Kohler, T. Zemojtel, M. Spielmann*, et al.* A Whole-Genome Analysis Framework for Effective Identification of Pathogenic Regulatory Variants in Mendelian Disease*.* *Am J Hum Genet*, 2016. **99**(3): p. 595-606.

12. Dayem Ullah, A.Z., J. Oscanoa, J. Wang, A. Nagano, N.R. Lemoine and C. Chelala. SNPnexus: assessing the functional relevance of genetic variation to facilitate the promise of precision medicine*.* *Nucleic Acids Res*, 2018. **46**(W1): p. W109-W113.

13. Raine, K.M., J. Hinton, A.P. Butler, J.W. Teague, H. Davies, P. Tarpey*, et al.* cgpPindel: Identifying Somatically Acquired Insertion and Deletion Events from Paired End Sequencing*.* *Curr Protoc Bioinformatics*, 2015. **52**: p. 15 7 1-12.

14. Gel, B., A. Diez-Villanueva, E. Serra, M. Buschbeck, M.A. Peinado and R. Malinverni. regioneR: an R/Bioconductor package for the association analysis of genomic regions based on permutation tests*.* *Bioinformatics*, 2016. **32**(2): p. 289-91.

15. Gel, B. and E. Serra. karyoploteR: an R/Bioconductor package to plot customizable genomes displaying arbitrary data*.* *Bioinformatics*, 2017. **33**(19): p. 3088-3090.

16. Nik-Zainal, S., P. Van Loo, D.C. Wedge, L.B. Alexandrov, C.D. Greenman, K.W. Lau*, et al.* The life history of 21 breast cancers*.* *Cell*, 2012. **149**(5): p. 994-1007.

17. Nik-Zainal, S., H. Davies, J. Staaf, M. Ramakrishna, D. Glodzik, X. Zou*, et al.* Landscape of somatic mutations in 560 breast cancer whole-genome sequences*.* *Nature*, 2016. **534**(7605): p. 47-54.

18. Alexandrov, L.B., J. Kim, N.J. Haradhvala, M.N. Huang, A.W. Tian Ng, Y. Wu*, et al.* The repertoire of mutational signatures in human cancer*.* *Nature*, 2020. **578**(7793): p. 94-101.

19. Rustad, E.H., F. Nadeu, N. Angelopoulos, B. Ziccheddu, N. Bolli, X.S. Puente*, et al.* mmsig: a fitting approach to accurately identify somatic mutational signatures in hematological malignancies*.* *Commun Biol*, 2021. **4**(1): p. 424.

20. Rabbie, R., N. Ansari-Pour, O. Cast, D. Lau, F. Scott, S.J. Welsh*, et al.* Multi-site clonality analysis uncovers pervasive heterogeneity across melanoma metastases*.* *Nat Commun*, 2020. **11**(1): p. 4306.

21. Alvarez-Prado, A.F., P. Perez-Duran, A. Perez-Garcia, A. Benguria, C. Torroja, V.G. de Yebenes*, et al.* A broad atlas of somatic hypermutation allows prediction of activation-induced deaminase targets*.* *J Exp Med*, 2018. **215**(3): p. 761-771.

22. Khodabakhshi, A.H., R.D. Morin, A.P. Fejes, A.J. Mungall, K.L. Mungall, M. Bolger-Munro*, et al.* Recurrent targets of aberrant somatic hypermutation in lymphoma*.* *Oncotarget*, 2012. **3**(11): p. 1308-19.
